# Neutrophil to lymphocyte ratio, platelet to lymphocyte ratio, and other hematological parameters in psoriasis patients

**DOI:** 10.1186/s12865-021-00454-4

**Published:** 2021-09-26

**Authors:** Wen-Ming Wang, Chao Wu, Yi-Meng Gao, Feng Li, Xiao-Ling Yu, Hong-Zhong Jin

**Affiliations:** Department of Dermatology, Peking Union Medical College Hospital, Chinese Academy of Medical Sciences and Peking Union Medical College, No.1 Shuaifuyuan Wangfujing Dongcheng District, Beijing, 100730 China

**Keywords:** Psoriasis, Neutrophil to lymphocyte ratio, Platelet to lymphocyte ratio, PASI

## Abstract

**Background:**

Psoriasis is a chronic immune‐mediated skin disorder. Systemic inflammation plays an important role in the pathogenesis of psoriasis.

**Methods:**

A total of 477 patients with psoriasis vulgaris (PsV, *n* = 347), generalized pustular psoriasis (GPP, *n* = 37), erythrodermic psoriasis (PsE, *n* = 45), arthritic psoriasis (PsA, *n* = 25) and mixed psoriasis (*n* = 23), and 954 healthy control subjects were included in the study. Demographic, clinical, and laboratory information were collected and compared between subgroups.

**Results:**

Compared with the healthy control group, patients with psoriasis had higher total white blood cell (WBC), neutrophil, platelet counts, neutrophil to lymphocyte ratio (NLR), and platelet to lymphocyte ratio (PLR), but lower hemoglobin (Hb) levels, lymphocyte and red blood cell (RBC) counts. NLR values in the PsV group were significantly lower than those in the GPP, PsE, and PsA groups, with GPP group being the highest. PLR values in the PsV group were significantly lower than those in the GPP, PsE, and PsA groups. There was no significant correlation between the psoriasis area severity index (PASI) score and either the NLR or PLR in the PsV group.

**Conclusions:**

Elevated NLR and PLR were associated with psoriasis and differed between subtypes, suggesting that they could be used as markers of systemic inflammation in psoriasis patients.

## Background

Psoriasis is a chronic, immune-mediated disorder that can involve the skin and/or joints [1]. Four clinical types of psoriasis are currently recognized: psoriasis vulgaris (PsV), pustular psoriasis (PP), erythrodermic psoriasis (PsE), and arthritic psoriasis (PsA) [2]. The psoriasis area severity index (PASI), which evaluates the degree of erythema, induration, and desquamation in the affected body areas, is one of the most commonly used scales to classify disease severity in patients with PsV, but the frequency of use is highly variable among clinicians [3]. Thus, obtaining uniformity in the diagnosis and assessment of psoriasis severity, especially for the subtypes, remains challenging.

Systemic inflammation plays an important role in the pathogenesis of psoriasis, and numerous inflammatory mediators have been implicated, including interleukins (e.g., IL-17, IL-1β), other cytokines and chemokines, and serum autoantibodies [4, 5]. However, there is an urgent need to identify better markers for the diagnosis and assessment of clinical severity and outcomes of psoriasis. Two potential markers are the NLR and PLR, which are easily and inexpensively measured and are relatively stable markers of subclinical inflammation [6, 7]. In addition, NLR and PLR have been identified as potential diagnostic and prognostic markers of other chronic inflammatory diseases and cancers [3, 8–10]. Recently, several studies have described the relationship between NLR, PLR, and psoriasis severity [11, 12].

Here, we analyzed the relationship between NLR, PLR, and disease severity in Chinese patients with psoriasis, and determined whether NLR and PLR differed in patients with four subtypes of psoriasis.

## Results

### Characteristics of psoriasis patients and control subjects

A total of 477 patients diagnosed with psoriasis (328 males, 149 females; mean age 43.46 ± 14.28 years) and 954 control subjects (656 males, 298 females; mean age 43.50 ± 14.30 years) were included in the study. Compared with the control group, patients with psoriasis had significantly higher WBC, neutrophil, and platelet counts, NLR, and PLR, but significantly lower lymphocyte and RBC counts and Hb levels (Table 1).

**Table 1:** Clinicopathological parameters in psoriasis patients and healthy control subjects

	Controls	Cases	*p* value
*Psoriasis*			
Sex (male/female)	656/298	328/149	1.0000
Age at enrollment	43.50 ± 14.30	43.46 ± 14.28	0.9697
WBC	6.31 ± 1.59	7.21 ± 2.31	< 0.0001
Lymphocyte	2.08 ± 0.60	1.81 ± 0.63	< 0.0001
Neutrophil	3.66 ± 1.19	4.63 ± 2.05	< 0.0001
RBC	4.94 ± 0.43	4.52 ± 0.55	< 0.0001
Hb	149.10 ± 13.62	138.68 ± 16.89	< 0.0001
PLT	231.69 ± 51.97	250.11 ± 85.24	0.0020
NLR	1.86 ± 0.74	2.89 ± 2.14	< 0.0001
PLR	118.82 ± 38.93	152.94 ± 75.88	< 0.0001

### Characteristics of patients with psoriasis subtypes

Of the 477 patients with psoriasis, 347, 37, 45 and 25 had PsV, GPP, PsE, and PsA, respectively. Compared with the control group, the PsV group had significantly higher WBC and neutrophil counts, NLR, and PLR, but significantly lower lymphocyte and RBC counts and Hb levels. Platelet counts did not differ between the PsV and control groups. The same differences were observed between the control group and each of the GPP, PsE, and PsA groups, with the exception that platelet counts were also significantly higher in the GPP, PsE, and PsA subgroups compared with the control group (Table 2).

**Table 2:** Clinicopathological parameters in patients stratified by psoriasis subtype

	Controls	Cases	*p* value
*PsV*			
Sex (male/female)	482/212	241/106	1.0000
Age at enrollment	43.11 ± 14.24	43.11 ± 14.23	> 0.9999
WBC	6.30 ± 1.60	6.84 ± 1.98	< 0.0001
Lymphocyte	2.14 ± 0.61	1.90 ± 0.66	< 0.0001
Neutrophil	3.61 ± 1.18	4.21 ± 1.62	< 0.0001
RBC	4.94 ± 0.43	4.62 ± 0.53	< 0.0001
Hb	149.10 ± 13.47	143.18 ± 14.54	< 0.0001
PLT	233.77 ± 51.86	233.35 ± 68.48	0.5899
NLR	1.78 ± 0.68	2.41 ± 1.21	< 0.0001
PLR	116.41 ± 37.08	134.03 ± 56.82	< 0.0001
*GPP*			
Sex (male/female)	40/34	20/17	1.0000
Age at enrollment	41.14 ± 14.25	41.24 ± 14.16	> 0.9999
WBC	6.50 ± 1.61	9.53 ± 2.99	< 0.0001
Lymphocyte	2.15 ± 0.57	1.52 ± 0.56	< 0.0001
Neutrophil	3.77 ± 1.19	7.10 ± 2.88	< 0.0001
RBC	4.93 ± 0.45	4.27 ± 0.37	< 0.0001
Hb	146.24 ± 13.28	128.86 ± 14.32	< 0.0001
PLT	240.41 ± 45.93	289.95 ± 102.14	0.0311
NLR	1.82 ± 0.61	5.90 ± 5.15	< 0.0001
PLR	118.29 ± 33.38	221.82 ± 126.01	< 0.0001
*PsE*			
Sex (male/female)	78/12	39/6	1.0000
Age at enrollment	49.47 ± 13.80	49.47 ± 13.88	> 0.9999
WBC	6.42 ± 1.69	7.14 ± 2.02	0.0448
Lymphocyte	2.16 ± 0.59	1.61 ± 0.45	< 0.0001
Neutrophil	3.69 ± 1.22	4.57 ± 1.72	0.0023
RBC	4.98 ± 0.41	4.22 ± 0.54	< 0.0001
Hb	151.40 ± 10.71	129.13 ± 15.27	< 0.0001
PLT	224.77 ± 48.53	271.02 ± 78.07	0.0003
NLR	1.77 ± 0.59	3.02 ± 1.34	< 0.0001
PLR	110.12 ± 33.51	182.99 ± 82.45	< 0.0001
*PsA*			
Sex (male/female)	30/20	15/10	1.0000
Age at enrollment	43.04 ± 14.16	43.04 ± 14.31	> 0.9999
WBC	5.97 ± 1.46	7.71 ± 2.68	0.0033
Lymphocyte	1.92 ± 0.56	1.59 ± 0.42	0.0092
Neutrophil	3.56 ± 1.06	5.38 ± 2.38	0.0003
RBC	4.84 ± 0.39	4.12 ± 0.57	<0.0001
Hb	146.36 ± 13.00	121.40 ± 21.49	<0.0001
PLT	214.42 ± 38.47	311.08 ± 134.54	<0.0001
NLR	1.96 ± 0.71	3.49 ± 1.56	<0.0001
PLR	119.44 ± 35.93	199.06 ± 74.24	<0.0001

### Comparison of hematological parameters between patients with different psoriasis subtypes

**Fig. 1:**
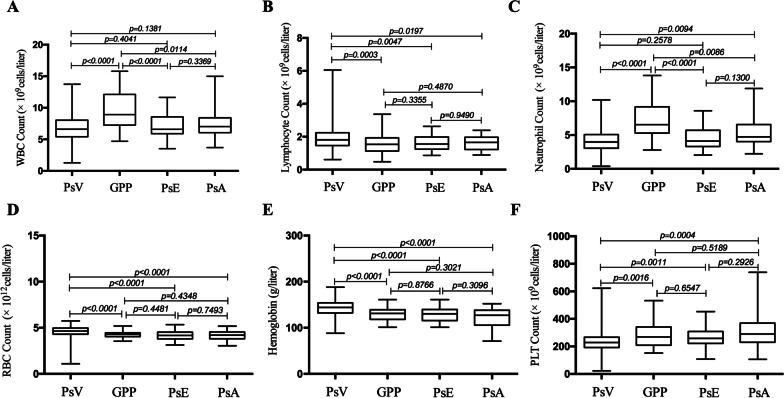
Hematological parameters in patients stratified by psoriasis subtype

WBC and neutrophil counts were significantly higher in the GPP group than in the PsV, PsE, and PsA groups, whereas lymphocyte and RBC counts and Hb levels were significantly higher in the PsV group than in the GPP, PsE, and PsA groups. These parameters were not significantly different between the GPP, PsE, and PsA groups. Platelet count was significantly lower in the PsV group than in the GPP, PsE, and PsA groups, and here too, there was no significant difference between the values in the GPP, PsE, and PsA groups (Fig. 1).

### Comparison of NLR and PLR between patients with different psoriasis subtypes

**Fig. 2:**
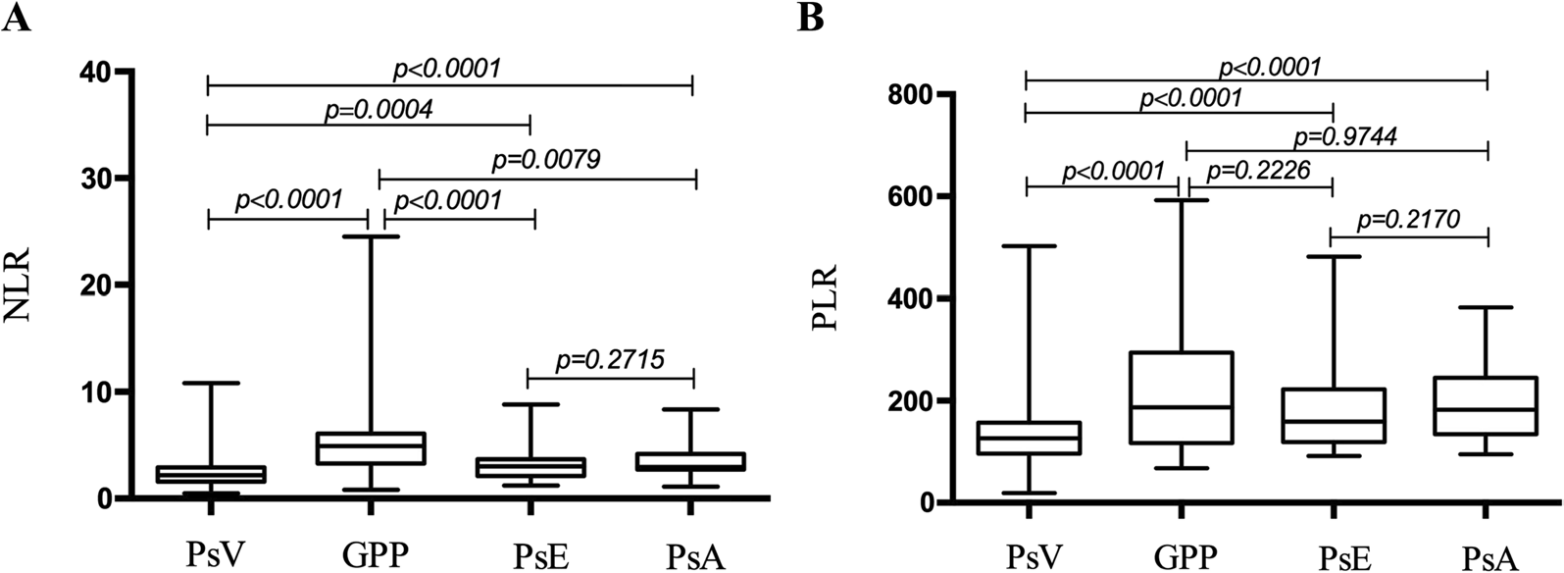
NLR and PLR in patients stratified by psoriasis subtype

The NLR was lowest in the PsV group and highest in the GPP group. The PLR was also lowest in the PsV group, but the ratio in the GPP, PsE, and PsA groups was comparable (Fig. 2).

### Correlation between PASI scores and hematological parameters in patients with PsV

PASI scores were recorded for 151 of the 347 (44%) PsV patients. Spearman’s correlation analysis indicated that the PASI score did not correlate significantly with any of the variables assessed (WBC, neutrophil, lymphocyte, platelet, or RBC counts, Hb level, NLR, or PLR; Table 3).

**Table 3:** Correlation between PASI scores and variables

	Correlation coefficient (*r*)	*p*
WBC	0.068	0.410
Neutrophil	0.003	0.966
Lymphocyte	− 0.042	0.606
RBC	− 0.044	0.589
Hb	0.014	0.860
Platelet	− 0.024	0.771
NLR	0.033	0.688
PLR	0.039	0.632

## Discussion

In this study of Chinese individuals, we found that the NLR and PLR were both significantly higher in patients with psoriasis than in healthy subjects. Our study is consistent with the results of previous studies of smaller patient populations, and we extended the findings to include a comprehensive and systematic evaluation of the association between psoriasis and multiple hematological parameters, including absolute blood cell counts, NLR, and PLR. Furthermore, we also compared these parameters in patients with the four subtypes of psoriasis. Neutrophils produce many inflammatory mediators and cytokines and are involved in a variety of systemic autoimmune diseases [13]. These cells also form so-called “neutrophil extracellular traps,” which are mesh-like arrays of DNA and protein aggregates that facilitate elimination of pathogens while sparing host cells. A previous study has demonstrated that neutrophil extracellular traps are more abundant in skin lesions and peripheral blood of psoriasis patients compared with healthy subjects [14, 15]. Th17 lymphocytes and their related cytokines are also considered to play crucial roles in psoriasis; however, neutrophils and mast cells are the major source of IL-17A in psoriatic skin lesions [16], substantiating the importance of neutrophils in the pathogenesis of psoriasis. Precious study showed that the levels of NLR in the psoriasis patients were higher than that in healthy controls [11, 17]. Polat M demonstrated that PASI scores was positively correlated with NLR values [11]. However, in accordance with our study, Ataseven A and colleagues found no correlation between NLR and PASI score [17]. A study included 316 psoriasis patients showed that elevated NLR was associated with severity of psoriasis (PASI scores) and non-calcified coronary artery burden. After 1 year treatment of biologics, NLR decreased and the degree of NLR decrease was related to the change of non-calcified coronary artery burden [10, 18]. Consistent with this study, another study demonstrated that PASI scores and NLR values of psoriasis patients can be decreased by systemic therapy, including narrow band ultraviolet B, acitretin, cyclosporine, methotrexate, adalimumab, etanercept, and ustekinumab. The degree of PASI scores and NLR values decrease was positively correlated [19]. However, study also found there was no significant difference of NLR and PLR between pre-treatment and after biologics treatment [20]. Therefore, studies also showed that elevated NLR values were positively correlated with increased PASI scores [12]. A meta-analysis included 1067 psoriasis patients and 799 healthy controls found that NLR were significantly higher in psoriasis patients than healthy controls. Furthermore, NLR and PLR values in patients with PASI > 10 was higher than that of patients with PASI < 10. But there were no association between NLR values and PASI scores [7, 21]. These studies indicate that NLR values were elevated in psoriasis patients and decreased after systemic therapy. But the results of relationship between NLR and the severity of psoriasis were inconsistent. Our study demonstrated that the neutrophil counts and NLR were significantly higher in all four psoriasis subtype groups compared with the control group, with the GPP group having the highest NLR. We assumed that reasons for inconsistent research results may include number of cases, disease duration, types of study (for example retrospective study or prospective study), other disease combined, racial and ethnic differences and so on. Overall, these studies suggest that NLR values were correlated with psoriasis, but may not the severity of psoriasis. In conclusion, whether neutrophil counts and NLR can be used as markers to distinguish between the disease subtypes, and the exact contribution of neutrophils to the pathogenesis of psoriasis, remain to be clarified.

Platelets, which are often elevated during infectious and inflammatory diseases [22], store a number of inflammatory cytokines and chemokines that play crucial roles in psoriasis, such as IL-1β and CXCL8 [23–25]. The observed increased abundance of platelets in psoriasis patients compared with control subjects is probably a consequence of chronic inflammation. PLR is another proposed indicator of systemic inflammation [26]. Precious study showed that PLR values in the psoriasis patients were higher compared to healthy controls [11, 17]. Furthermore, PLR values can be downregulated after biologics therapy [7, 20]. A meta-analysis also showed that PLR values were significantly higher in psoriasis patients than that in healthy controls [21].

In agreement with previous studies [7, 11], we found that both peripheral blood platelet counts and PLR are significantly higher in patients with each of the four subtypes of psoriasis compared with the control subjects. The PLR was lowest in the PsV group and similar in the GPP, PsE, and PsA groups. Although these data demonstrate that platelet counts and PLR are increased in psoriasis patients, especially in the most severe subtype, the underlying mechanisms are unclear.

Conflicting data have been reported about the relationship between psoriasis and both RBC and Hb. Several studies have reported no significant difference in Hb levels between psoriasis patients and healthy subjects [12, 27], whereas we found that RBC and Hb were both significantly decreased in all of the psoriasis subgroups compared with the control group. The highest values were observed in the PsV group, with the GPP, PsE, and PsA groups having similar Hb levels. A previous study demonstrated that erythrocyte membrane fluidity was lower in psoriasis patients than in healthy subjects. Moreover, psoriasis patients have been shown to have decreased levels of the antioxidant enzymes superoxide dismutase and catalase and, conversely, elevated levels of the oxidative stress marker malondialdehyde, compared with control subjects [28]. Thus, we hypothesize that the reduction in RBC count and Hb level observed in our cohort of psoriasis patients may be related to neutrophil activation and oxidative stress [29].

Earlier studies found that NLR and PLR reflect disease activity in psoriasis patients [7, 12]; however, we failed to detect a positive association between PASI scores and either NLR or PLR. There are at least two explanations for this apparent discrepancy. For example, NLR and PLR may reflect the inflammatory status of psoriasis patients, but not the disease severity. Alternatively, the PASI may not be sufficiently sensitive to disease symptoms, particularly those of mild to moderate severity and those affecting the nails. Moreover, the index does not take into account the disease impact on quality of life and comorbidities, both of which may influence the ratios [30]. Clearly, further studies are needed to clarify the relationship between PASI scores, PLR, and NLR.

Finally, our study may have limitations, including it was conducted in a single tertiary medical center and was a cross-sectional study, PASI scores of some patients were not calculated, both the patients and healthy controls who combined malignancy except hematological malignancies were not excluded. These may lead to the patients were biased to having higher disease severity and could not fully represent the features of psoriasis patients in China.

## Conclusion

In conclusion, our results highlight the significant differences in NLR and PLR among psoriasis patients. Neutrophil counts and NLR were significantly higher in psoriasis group compared with the control group, with the GPP group having the highest NLR. In addition, platelet counts and PLR are increased in psoriasis patients, especially in the most severe subtype. However, further work will be necessary to resolve the many remaining questions about the clinical relevance of NLR and PLR in psoriasis and their potential utility as diagnostic and/or prognostic markers.

## Methods

This study was a hospital-based cross-sectional study. This retrospective study enrolled 477 adult patients with psoriasis who had visited the Department of Dermatology at Peking Union Medical College Hospital, which is a tertiary medical center, between 2005 and 2015. All patients were diagnosed based on clinical or histopathological manifestations of psoriasis. Patients were excluded if they had received any systemic treatment, such as corticosteroids, acitretin, methotrexate, or biologics, during 2 months preceding enrollment. Patients who had hematological malignancies were also excluded. The control group consisted of 954 age- and sex-matched healthy volunteers who were recruited from the Department of Physical Examination Center of Peking Union Medical College Hospital in 2015. They had no hepatic disorders, kidney dysfunction, infectious diseases or hematological malignancies. The protocol was approved by the institutional review board of Peking Union Medical College Hospital. Written informed consent was not required for this retrospective study.

### Laboratory values and clinical assessment

Demographic, clinical, and laboratory data were obtained from electronic or handwritten medical records. The following parameters were extracted: WBC, neutrophil, lymphocyte, RBC, and platelet counts, and Hb levels. In addition, NLR and PLR were calculated and the PASI scoring system was used to assess the severity of PsV.

### Statistical analysis

Continuous data are described as the mean ± standard deviation. Differences between groups were assessed using the Mann–Whitney non-parametric test, where appropriate. Correlations were evaluated using Spearman’s correlation coefficient. A *p* value of < 0.05 was considered statistically significant. All analyses were performed using SPSS version 25.0 (IBM, Armonk, NY, USA) and Prism version 6.0 (GraphPad Software, San Diego, CA, USA).

## Data Availability

The datasets used and/or analysed during the current study are not publicly available due the privacy of patients but are available from the corresponding author on reasonable request.

## Abbreviations

PsV: Psoriasis vulgaris
GPP: Generalized pustular psoriasis
PsE: Erythrodermic psoriasis
PsA: Arthritic psoriasis
WBC: White blood cell
NLR: Neutrophil to lymphocyte ratio
PLR: Platelet to lymphocyte ratio
Hb: Hemoglobin
RBC: Red blood cell
PASI: Psoriasis area severity index
